# Supplementary material for: Dynalign II: common secondary structure prediction for RNA homologs with domain insertions
Source: Nucleic Acids Res. 2014 Nov 21;42(22):13939–48. doi: 10.1093/nar/gku1172 (PMC4267632; doi:10.1093/nar/gku1172)
Supplement: SUPPLEMENTARY DATA [file supp_gku1172_nar-02021-z-2014-File012.zip › manual/javadoc/RNAstructure_java_drawing/source/dialogs/PlotDialog.html]

PlotDialog


JavaScript is disabled on your browser.


- Overview
- Package
- Class
- Tree
- Index
- Help

- Prev Class
- Next Class

- Frames
- No Frames

- All Classes

- Summary:
- Nested |
- Field |
- Constr |
- Method

- Detail:
- Field |
- Constr |
- Method


RNAstructure\_java\_drawing.source.dialogs

## Class PlotDialog

- java.lang.Object
- - java.awt.Component
  - - java.awt.Container
    - - java.awt.Window
      - - java.awt.Dialog
        - - javax.swing.JDialog
          - - RNAstructure\_java\_drawing.source.dialogs.ImageDialog
            - - RNAstructure\_java\_drawing.source.dialogs.PlotDialog

- All Implemented Interfaces:
  :   ImageObserver, MenuContainer, Printable, Serializable, Accessible, RootPaneContainer, WindowConstants

  ---

    

  ```
  public class PlotDialog
  extends ImageDialog
  ```

  A dialog that holds dot plot images.

  See Also:
  :   Serialized Form

- - ### Nested Class Summary

    - ### Nested classes/interfaces inherited from class javax.swing.JDialog

      `JDialog.AccessibleJDialog`
    - ### Nested classes/interfaces inherited from class java.awt.Dialog

      `Dialog.AccessibleAWTDialog, Dialog.ModalExclusionType, Dialog.ModalityType`
    - ### Nested classes/interfaces inherited from class java.awt.Window

      `Window.AccessibleAWTWindow, Window.Type`
    - ### Nested classes/interfaces inherited from class java.awt.Container

      `Container.AccessibleAWTContainer`
    - ### Nested classes/interfaces inherited from class java.awt.Component

      `Component.AccessibleAWTComponent, Component.BaselineResizeBehavior, Component.BltBufferStrategy, Component.FlipBufferStrategy`
  - ### Field Summary

    Fields

    | Modifier and Type | Field and Description |
    | --- | --- |
    | `private DotPlotBackend` | `backend` The back end object that reads data for dot plots. |
    | `private int` | `plotLength` The length of the plot. |
    | `private static long` | `serialVersionUID` |
    | `private int` | `strand` The strand being drawn. |

    - ### Fields inherited from class RNAstructure\_java\_drawing.source.dialogs.ImageDialog

      `error, file, mainPanel, maxBoundX, maxBoundY, preferredDialogWidth, scale`
    - ### Fields inherited from class javax.swing.JDialog

      `accessibleContext, rootPane, rootPaneCheckingEnabled`
    - ### Fields inherited from class java.awt.Dialog

      `DEFAULT_MODALITY_TYPE`
    - ### Fields inherited from class java.awt.Component

      `BOTTOM_ALIGNMENT, CENTER_ALIGNMENT, LEFT_ALIGNMENT, RIGHT_ALIGNMENT, TOP_ALIGNMENT`
    - ### Fields inherited from interface java.awt.print.Printable

      `NO_SUCH_PAGE, PAGE_EXISTS`
    - ### Fields inherited from interface javax.swing.WindowConstants

      `DISPOSE_ON_CLOSE, DO_NOTHING_ON_CLOSE, EXIT_ON_CLOSE, HIDE_ON_CLOSE`
    - ### Fields inherited from interface java.awt.image.ImageObserver

      `ABORT, ALLBITS, ERROR, FRAMEBITS, HEIGHT, PROPERTIES, SOMEBITS, WIDTH`
  - ### Constructor Summary

    Constructors

    Constructor and Description

    `PlotDialog(String file)`

    File Constructor.

    `PlotDialog(String file,
    int strand)`

    Strand Constructor.
  - ### Method Summary

    Methods

    | Modifier and Type | Method and Description |
    | --- | --- |
    | `protected void` | `createDrawnImage(Graphics2D g2)` Create the image drawn on this panel. |
    | `Integer[]` | `getColors()` Get the range of colors allowed in this dialog. |
    | `Double[]` | `getCurrentBounds()` Get the current bounds allowed in this dialog. |
    | `Double[]` | `getDefaultBounds()` Get the default bounds allowed in this dialog. |
    | `protected void` | `readImageData()` Read image data from a particular file. |
    | `void` | `setColors(int colors)` Set the number of colors in the plot on this dialog. |
    | `void` | `setRange(double minimum, double maximum)` Set the range of the plot on this dialog. |
    | `void` | `writePostscriptFile(String outFile)` Write a Postscript file from the plot on this dialog. |
    | `boolean` | `writeStructuresFile(String outFile, boolean showStructures)` Write a probable structures file from the plot on this dialog. |
    | `void` | `writeSVGFile(String outFile)` Write an SVG file from the plot on this dialog. |
    | `void` | `writeTextFile(String outFile)` Write a text file from the plot on this dialog. |

    - ### Methods inherited from class RNAstructure\_java\_drawing.source.dialogs.ImageDialog

      `buildLegend, createDefaultMenuBar, getFile, getScale, isError, print, printPanel, refresh, setScale, setTopCaption, viewDialog, viewZoomDialog, zoomImage`
    - ### Methods inherited from class javax.swing.JDialog

      `addImpl, createRootPane, dialogInit, getAccessibleContext, getContentPane, getDefaultCloseOperation, getGlassPane, getGraphics, getJMenuBar, getLayeredPane, getRootPane, getTransferHandler, isDefaultLookAndFeelDecorated, isRootPaneCheckingEnabled, paramString, processWindowEvent, remove, repaint, setContentPane, setDefaultCloseOperation, setDefaultLookAndFeelDecorated, setGlassPane, setJMenuBar, setLayeredPane, setLayout, setRootPane, setRootPaneCheckingEnabled, setTransferHandler, update`
    - ### Methods inherited from class java.awt.Dialog

      `addNotify, getModalityType, getTitle, hide, isModal, isResizable, isUndecorated, setBackground, setModal, setModalityType, setOpacity, setResizable, setShape, setTitle, setUndecorated, setVisible, show, toBack`
    - ### Methods inherited from class java.awt.Window

      `addPropertyChangeListener, addPropertyChangeListener, addWindowFocusListener, addWindowListener, addWindowStateListener, applyResourceBundle, applyResourceBundle, createBufferStrategy, createBufferStrategy, dispose, getBackground, getBufferStrategy, getFocusableWindowState, getFocusCycleRootAncestor, getFocusOwner, getFocusTraversalKeys, getIconImages, getInputContext, getListeners, getLocale, getModalExclusionType, getMostRecentFocusOwner, getOpacity, getOwnedWindows, getOwner, getOwnerlessWindows, getShape, getToolkit, getType, getWarningString, getWindowFocusListeners, getWindowListeners, getWindows, getWindowStateListeners, isActive, isAlwaysOnTop, isAlwaysOnTopSupported, isAutoRequestFocus, isFocusableWindow, isFocusCycleRoot, isFocused, isLocationByPlatform, isOpaque, isShowing, isValidateRoot, pack, paint, postEvent, processEvent, processWindowFocusEvent, processWindowStateEvent, removeNotify, removeWindowFocusListener, removeWindowListener, removeWindowStateListener, reshape, setAlwaysOnTop, setAutoRequestFocus, setBounds, setBounds, setCursor, setFocusableWindowState, setFocusCycleRoot, setIconImage, setIconImages, setLocation, setLocation, setLocationByPlatform, setLocationRelativeTo, setMinimumSize, setModalExclusionType, setSize, setSize, setType, toFront`
    - ### Methods inherited from class java.awt.Container

      `add, add, add, add, add, addContainerListener, applyComponentOrientation, areFocusTraversalKeysSet, countComponents, deliverEvent, doLayout, findComponentAt, findComponentAt, getAlignmentX, getAlignmentY, getComponent, getComponentAt, getComponentAt, getComponentCount, getComponents, getComponentZOrder, getContainerListeners, getFocusTraversalPolicy, getInsets, getLayout, getMaximumSize, getMinimumSize, getMousePosition, getPreferredSize, insets, invalidate, isAncestorOf, isFocusCycleRoot, isFocusTraversalPolicyProvider, isFocusTraversalPolicySet, layout, list, list, locate, minimumSize, paintComponents, preferredSize, print, printComponents, processContainerEvent, remove, removeAll, removeContainerListener, setComponentZOrder, setFocusTraversalKeys, setFocusTraversalPolicy, setFocusTraversalPolicyProvider, setFont, transferFocusDownCycle, validate, validateTree`
    - ### Methods inherited from class java.awt.Component

      `action, add, addComponentListener, addFocusListener, addHierarchyBoundsListener, addHierarchyListener, addInputMethodListener, addKeyListener, addMouseListener, addMouseMotionListener, addMouseWheelListener, bounds, checkImage, checkImage, coalesceEvents, contains, contains, createImage, createImage, createVolatileImage, createVolatileImage, disable, disableEvents, dispatchEvent, enable, enable, enableEvents, enableInputMethods, firePropertyChange, firePropertyChange, firePropertyChange, firePropertyChange, firePropertyChange, firePropertyChange, firePropertyChange, firePropertyChange, firePropertyChange, getBaseline, getBaselineResizeBehavior, getBounds, getBounds, getColorModel, getComponentListeners, getComponentOrientation, getCursor, getDropTarget, getFocusListeners, getFocusTraversalKeysEnabled, getFont, getFontMetrics, getForeground, getGraphicsConfiguration, getHeight, getHierarchyBoundsListeners, getHierarchyListeners, getIgnoreRepaint, getInputMethodListeners, getInputMethodRequests, getKeyListeners, getLocation, getLocation, getLocationOnScreen, getMouseListeners, getMouseMotionListeners, getMousePosition, getMouseWheelListeners, getName, getParent, getPeer, getPropertyChangeListeners, getPropertyChangeListeners, getSize, getSize, getTreeLock, getWidth, getX, getY, gotFocus, handleEvent, hasFocus, imageUpdate, inside, isBackgroundSet, isCursorSet, isDisplayable, isDoubleBuffered, isEnabled, isFocusable, isFocusOwner, isFocusTraversable, isFontSet, isForegroundSet, isLightweight, isMaximumSizeSet, isMinimumSizeSet, isPreferredSizeSet, isValid, isVisible, keyDown, keyUp, list, list, list, location, lostFocus, mouseDown, mouseDrag, mouseEnter, mouseExit, mouseMove, mouseUp, move, nextFocus, paintAll, prepareImage, prepareImage, printAll, processComponentEvent, processFocusEvent, processHierarchyBoundsEvent, processHierarchyEvent, processInputMethodEvent, processKeyEvent, processMouseEvent, processMouseMotionEvent, processMouseWheelEvent, remove, removeComponentListener, removeFocusListener, removeHierarchyBoundsListener, removeHierarchyListener, removeInputMethodListener, removeKeyListener, removeMouseListener, removeMouseMotionListener, removeMouseWheelListener, removePropertyChangeListener, removePropertyChangeListener, repaint, repaint, repaint, requestFocus, requestFocus, requestFocusInWindow, requestFocusInWindow, resize, resize, revalidate, setComponentOrientation, setDropTarget, setEnabled, setFocusable, setFocusTraversalKeysEnabled, setForeground, setIgnoreRepaint, setLocale, setMaximumSize, setName, setPreferredSize, show, size, toString, transferFocus, transferFocusBackward, transferFocusUpCycle`
    - ### Methods inherited from class java.lang.Object

      `clone, equals, finalize, getClass, hashCode, notify, notifyAll, wait, wait, wait`

- - ### Field Detail


    - #### backend

      ```
      private DotPlotBackend backend
      ```

      The back end object that reads data for dot plots.


    - #### plotLength

      ```
      private int plotLength
      ```

      The length of the plot.


    - #### serialVersionUID

      ```
      private static final long serialVersionUID
      ```

      See Also:
      :   Constant Field Values


    - #### strand

      ```
      private int strand
      ```

      The strand being drawn.
  - ### Constructor Detail


    - #### PlotDialog

      ```
      public PlotDialog(String file)
      ```

      File Constructor.

      Parameters:
      :   `file` - The dot plot data file to draw.


    - #### PlotDialog

      ```
      public PlotDialog(String file,
                int strand)
      ```

      Strand Constructor.

      Parameters:
      :   `file` - The dot plot data file to draw.
      :   `strand` - The strand from the data file to draw.
  - ### Method Detail


    - #### createDrawnImage

      ```
      protected void createDrawnImage(Graphics2D g2)
      ```

      **Description copied from class: `ImageDialog`**

      Create the image drawn on this panel.

      **Specified by:**
      :   `createDrawnImage` in class `ImageDialog`

      Parameters:
      :   `g2` - The graphics context.


    - #### getColors

      ```
      public Integer[] getColors()
      ```

      Get the range of colors allowed in this dialog.

      Returns:
      :   The color range.


    - #### getCurrentBounds

      ```
      public Double[] getCurrentBounds()
      ```

      Get the current bounds allowed in this dialog.

      Returns:
      :   The current bounds.


    - #### getDefaultBounds

      ```
      public Double[] getDefaultBounds()
      ```

      Get the default bounds allowed in this dialog.

      Returns:
      :   The default bounds.


    - #### readImageData

      ```
      protected void readImageData()
      ```

      **Description copied from class: `ImageDialog`**

      Read image data from a particular file.

      **Specified by:**
      :   `readImageData` in class `ImageDialog`


    - #### setColors

      ```
      public void setColors(int colors)
      ```

      Set the number of colors in the plot on this dialog.

      Parameters:
      :   `colors` - The number of colors to set.


    - #### setRange

      ```
      public void setRange(double minimum,
                  double maximum)
      ```

      Set the range of the plot on this dialog.

      Parameters:
      :   `minimum` - The lower bound of the range.
      :   `maximum` - The upper bound of the range.


    - #### writePostscriptFile

      ```
      public void writePostscriptFile(String outFile)
      ```

      Write a Postscript file from the plot on this dialog.

      Parameters:
      :   `outFile` - The file to write.


    - #### writeStructuresFile

      ```
      public boolean writeStructuresFile(String outFile,
                                boolean showStructures)
      ```

      Write a probable structures file from the plot on this dialog.

      Parameters:
      :   `outFile` - The file to write.
      :   `showStructures` - True if structures should be shown, false if not.

      Returns:
      :   True if structures were written correctly, false
          if not.


    - #### writeSVGFile

      ```
      public void writeSVGFile(String outFile)
      ```

      Write an SVG file from the plot on this dialog.

      Parameters:
      :   `outFile` - The file to write.


    - #### writeTextFile

      ```
      public void writeTextFile(String outFile)
      ```

      Write a text file from the plot on this dialog.

      Parameters:
      :   `outFile` - The file to write.


- Overview
- Package
- Class
- Tree
- Index
- Help

- Prev Class
- Next Class

- Frames
- No Frames

- All Classes

- Summary:
- Nested |
- Field |
- Constr |
- Method

- Detail:
- Field |
- Constr |
- Method
